# Morphological feature and mapping inflammation in classified carotid plaques in symptomatic and asymptomatic patients: A hybrid ^18^F-FDG PET/MR study

**DOI:** 10.3389/fnins.2023.1144248

**Published:** 2023-03-21

**Authors:** Yue Zhang, Bixiao Cui, Hongwei Yang, Jie Ma, Yu Yang, Bin Yang, Yan Ma, Liqun Jiao, Xiang Li, Jie Lu

**Affiliations:** ^1^Department of Radiology and Nuclear Medicine, Xuanwu Hospital, Capital Medical University, Beijing, China; ^2^Beijing Key Laboratory of Magnetic Resonance Imaging and Brain Informatics, Beijing, China; ^3^Department of Neurosurgery, Xuanwu Hospital, Capital Medical University, Beijing, China; ^4^Division of Nuclear Medicine, Department of Biomedical Imaging and Image-Guided Therapy, Medical University of Vienna, Vienna, Austria

**Keywords:** hybrid ^18^F-FDG PET/MR, atherosclerosis, vulnerable plaque, carotid arteries, symptomatic, asymptomatic

## Abstract

**Purpose:**

To investigate morphological and inflamed-metabolism features of carotid atherosclerotic plaques between symptomatic and asymptomatic patients with hybrid ^18^F-FDG PET/MR imaging.

**Methods:**

A total of 20 symptomatic and 20 asymptomatic patients with carotid plaques underwent hybrid ^18^F-FDG PET/MR scans. American heart association (AHA) lesion types were classified, and plaque compositions were further determined on consecutive MRI axial sections in both carotid arteries. ^18^F-FDG uptake in carotid arteries was quantified using region of interest (ROI) methods based on maximum standardized uptake values (SUVmax) and target-to-background ratio (TBR) on corresponding positron emission tomography (PET) images.

**Results:**

A total of seventy-one carotid plaques were quantified. AHA type VI was the most common (23, 32.4%), and the region of carotid bifurcation was the most common place presenting lesions (32, 45.1%). Compared with the asymptomatic group, the prevalence of high-risk features including plaque burden, lumen stenosis, maximum necrotic core area, and maximum intra-plaque hemorrhage area increased in the symptomatic group. Carotid TBR values of plaque in symptomatic group (TBR = 2.56 ± 0.34) was significantly higher than that in asymptomatic group (TBR = 1.57 ± 0.14) (*P* < 0.05). hs-CRP is an independent risk factor for the stability of carotid plaque. The correlation between normalized wall index (NWI) and TBR values was significantly positive in both the symptomatic and the asymptomatic groups (*P* < 0.01), and both NWI and TBR were significantly correlated with the level of hs-CRP (*P* < 0.01).

**Conclusion:**

Integrated ^18^F-FDG PET/MR scans presented distinct risk features between symptomatic and asymptomatic patients. Hybrid ^18^F-FDG PET/MR systems combined with clinical serum hs-CRP may help distinguish vulnerable carotid plaques.

## Introduction

Stroke is one of the leading causes of death in Asia, especially in China (Liu et al., 2011; GBD 2016 Stroke Collaborators, 2019). In estimation, around 18 to 25% of ischemic strokes were caused by carotid plaques (Feigin et al., 2014; Saba et al., 2019). Plaque rupture is identified in about 70% of endarterectomy specimens of patients presenting with ischemic stroke and is considered a critical factor for arterial thromboembolism (Naghavi et al., 2003). Vulnerable plaque is a special type of plaque that is prone to rupture and lead to thromboembolism in the progress of plaque, and also includes some non-ruptured plaques that are prone to cause acute cerebrovascular events. In Naghavi et al. (2003), researchers reached a consensus that vulnerable plaque refers to “all dangerous plaque with rupture tendency, easy to form thrombus and/or rapid progress,” which is closely related to the occurrence of clinical ischemic stroke. The typical pathological manifestations of vulnerable plaque include large lipid-rich necrotic core (LRNC), thin or ruptured fibrous cap, intraplaque hemorrhage (IPH), infiltration of a large number of inflammatory cells, formation of new blood vessels, calcification of plaque surface, and ulcer of plaque surface. Among them, the first three are the main characteristics of vulnerable plaque (Gijsen et al., 2015; Daemen et al., 2016). In addition, the plaque with high load could frequently cause plaque rupture due to its remodeling toward the lumen. Therefore, early detection of vulnerable plaque is of great significance to the risk stratification and clinical intervention of carotid atherosclerotic plaque patients, so that to reduce the incidence of ischemic stroke.

Accordingly, non-invasive detection of ruptured plaque with imaging techniques are playing increasingly important roles in identifying the cause of stroke and intermittent cerebral ischemia in symptomatic patients and improving risk stratification in asymptomatic patients. The traditional vascular imaging techniques include color Doppler ultrasound, DSA, CTA, and MRA. The above examination techniques can only observe the extent of carotid artery stenosis, and cannot clearly display the internal components of the plaque, yielding limitations in the assessment of plaque stability (Daemen et al., 2016). The continuously developed high resolution (HR)-MRI technology in recent years can not only display the stenosis of the vascular lumen but also directly observe the structure of the vessel wall, potentially providing qualitative and quantitative analysis of the plaque (Fabiano et al., 2008). On the other hand, ^18^F-fluorodeoxyglucose positron emission tomography (^18^F-FDG-PET) is a molecular imaging technology, which could be used to observe the metabolism of macrophages in atherosclerotic plaques, so as to judge the inflammatory reaction in plaque and identify vulnerable plaque from the molecular level (Rudd et al., 2002; Davies et al., 2010; Ng et al., 2023).

As the lesion of carotid artery plaque is small, the accuracy of the results will be affected by slight registration error when the image fusion is performed by MRI and ^18^F-FDG-PET. With the application of integrated ^18^F-FDG-PET/MR, MRI, and ^18^F-FDG-PET synchronous scans could be completed simultaneously to achieve accurate fusion of the two images, which can more accurately assess the stability of plaque by providing plaque morphology and molecular level information (Mootaz et al., 2015; Li et al., 2016). In this study, we assessed the potential role of a simultaneous PET/MR system in patients with carotid atherosclerotic plaques, explored the differences between the components of carotid plaques in different MRI AHA types and TBR values, studied the correlation between the imaging biomarkers with clinical indicators, and compared the degree of inflammatory reaction between different plaques.

## Materials and methods

### Patients

A total of 40 patients with carotid atherosclerotic plaques admitted to the Department of Neurosurgery, Xuanwu Hospital, Capital Medical University, Beijing, China, from October 2018 to October 2020, were enrolled in this study, divided into symptomatic group and asymptomatic group: (1) the symptomatic group with neurological deficit symptoms in recent 3 months, including transient amaurosis, transient ischemic attack, ischemic stroke, excluding other causes, carotid atherosclerotic plaque was considered as the responsible lesion; (2) the asymptomatic group without ischemic symptoms, ultrasound found carotid artery plaque and intima-media thickness >2 mm. The exclusion criteria included (1) patients with carotid artery occlusion; (2) contraindications of PET/MR examination; (3) other intracranial organic lesions founded by MRI; (4) recent infection, malignant tumor, immune deficiency disease, multiple system disease, including severe liver function, renal dysfunction and blood system disease, etc; (5) unsuccessful PET/MR examination. The patients were divided into two groups according to their symptoms: asymptomatic group of 20 cases, symptomatic conservative treatment group of 20 cases. The gender, age, height, weight, blood pressure, and the serum levels of total cholesterol, triglyceride, low density lipoprotein, fasting blood glucose, homocysteinemia, hypersensitive C-reactive protein (hs-CRP), and glycosylated hemoglobin, and the related medical history were collected. All subjects provided written informed consent and the study was approved by the ethics committee of Xuanwu Hospital and conducted in accordance with the Declaration of Helsinki.

### PET/MR acquisition

All images were acquired on a hybrid TOF PET/MR system (SIGNA, GE Healthcare, WI, USA). A 19-channel head and neck union coil was used for MR imaging.

#### PET acquisition protocol

All patients were asked to fast for at least 6 h before ^18^F-FDG PET imaging. Tracer injection and imaging was performed only if fasting glucose was lower than 8.0 mmol/l. ^18^F-FDG was injected intravenously, and the injection dosage was calculated based on patients’ body weight (3.7 MBq/kg). The patient’s neck was placed in a support device on the examination table and covered with dedicated carotid MRI coils. Imaging started at 130 ± 15 min after tracer injection on a fully integrated PET/MR scanner. The field of view for positron emission tomography (PET) acquisition was centered on the carotid bifurcations, identified by TOF MR angiography. PET was then acquired in 3-D mode for 10 min in a single bed position. The PET bed position included a simultaneous 18-s-2-point Dixon scan for MRI (Martinez-Möller et al., 2009), which would be used for MR attenuation correction (MRAC). Data were corrected for random, dead time, scatter and attenuation based on the μ-maps extracted from the Dixon images. Reconstruction was performed with time-of-flight (TOF), point spread function (PSF), ordered subset expectation maximization (OSEM) algorithm (2 iterations, 28 subsets) and a 3-mm cut-off filter. Reconstructed PET images were reconstructed with a 35 mm× 35 cm field of view and a 192 × 192 matrix. Spatial resolution of the reconstructed PET images was previously determined to be 4.3 mm at 1 cm from the scanner’s isocenter and 5.0 mm at 10 cm in the transverse direction in full-width at half-maximum (Delso et al., 2011).

#### MRI acquisition protocol

All subjects were imaged using a previously published multiple sequence protocol (Saam et al., 2009) [TOF MR angiography, axial fat suppressed pre- and post-contrast black-blood T1-weighted imaging (T1WI) and T2-weighted imaging (T2WI) sequences; in-plane resolution 0.5 mm× 0.5 mm with the 3-T MRI system]. Parallel acquisition technique (PAT) was used for all sequences with an acceleration factor of 2. Imaging times for TOF, T1W and T2W images were 3.0, 4.0, and 4.0 min, respectively, resulting in a total scan time of 15 min. Gadolinium-DTPA-BMA (gadopentetate, Bayer, Leverkusen, Germany) of 0.1 mmol/kg (0.1 ml/kg) was given at a rate of 2.0 ml/s. Post-contrast T1WI was performed approximately 5 min after intravenous injection of the contrast agent.

### Data analysis

All integrated PET/MR images are completed using the post-processing workstation GE AW4.7 workstation and MR-Plaque View (VP Diagnostics, Seattle, WA, USA) (Brinjikji et al., 2018). All images interpretation work were jointly completed by two imaging physicians with more than 3 years of experience in HR-MRI and PET image interpretation of carotid plaque separately. The image interpretation personnel followed the blind principle for the clinical information of all subjects.

### Analysis of FDG PET images

PET images were analyzed by two nuclear medicine physician (BC. and JM.) blinded to clinical status and MRI. All images were reconstructed on a GE AW4.7 workstation. Matching of both data sets was considered as correct when neck contours of PET and MR images were perfectly aligned (Hutton et al., 2002; Hyafil et al., 2016). Circular regions of interest (ROIs) were placed on axial adjacent PET images of both carotid arteries (12 axial sections) identified using the MRA TOF images. Regions where FDG uptake located in adjacent lymph nodes. Maximum standard uptake values (SUVmax) calculated as decay-corrected tissue radioactivity divided by body weight and injected dose were recorded in ROIs (Hyafil et al., 2016). Tissue to background ratio (TBR) was calculated as the ratio of SUVmax and the background venous activity measured as SUVmean in the internal jugular veins (Rudd et al., 2008). In addition, mean TBRs for each arterial territory were calculated as the average of TBR values throughout the entire segment of the carotid artery present in the field of view (Huet et al., 2015; Hyafil et al., 2016).

### Analysis of MR images

**FIGURE 1:**
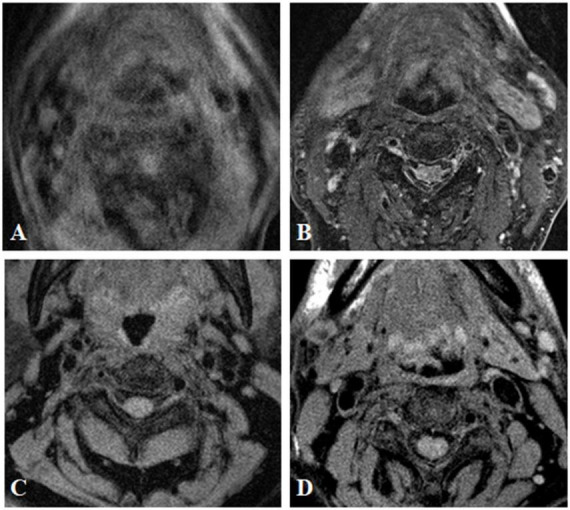
MR image quality evaluation of carotid plaque. The image quality of transverse axial T2WI showed carotid plaque was divided into grade 1 **(A)** grade 2 **(B)** grade 3 **(C)**, and grade 4 **(D)**.

Magnetic resonance imaging data were independently analyzed by two experienced radiologists (YZ. and HY.) who were blinded to the clinical status and PET diagnosis results. Image quality was assessed on a 4-point Likert scale (1 = worst; 4 = best) (Figure 1 and Table 1), and images with a value ≥2 were included for further evaluation (Hyafil et al., 2016). Atherosclerotic plaques in both carotid arteries were analyzed and classified slice by slice according to the modified criteria of the AHA (Cai et al., 2002). Area measurements of the lumen, wall, outer wall and tissue components were obtained using a custom designed semiautomatic image analysis tool named MR-Plaque View (Rudd et al., 2008). Tissue components (LR/NC, calcification and hemorrhage), fibrous cap status (thick vs. thin vs. ruptured), hemorrhage type [type I (early subacute) vs. type II (late subacute)] and thrombus presence (Hatsukami et al., 2000; Kampschulte et al., 2004; Saam et al., 2005; Hyafil et al., 2016).

**TABLE 1:** 4-point Likert criteria of carotid plaque MRI.

Grade	Criteria
1	Blurred, the lumen and the outside of blood vessels are not clear, and obvious motion artifacts
2	Wall of the tube was visible, but the contour of the wall or lumen and blood vessel was not clear, and there were many motion artifacts
3	Wall of the tube was clear, but the contour of lumen and the outside of blood vessel was fuzzy, and the artifacts were few, which did not affect the diagnosis
4	Wall structure, lumen and vascular contour were clearly displayed without obvious moving artifacts

### Statistical analysis

Categorical variables are presented as absolute and relative frequencies; continuous variables are presented as mean ± standard deviation (SD). Paired Student’s *t*-test was used to compare the differences between continuous variables and Fisher’s exact was used to test to determine differences between categorical variables. A two-sided Kruskal–Wallis test was used to compare the plaque composition of each axis of carotid arteries. A Mann–Whitney *U*-test was used to compare the difference between TBR values of different plaque components. The clinical risk factors of vulnerable plaque of carotid artery were evaluated by ordinal multi-classification logistic regression analysis, and the correlation between the two variables was analyzed by Spearman parameter test. All tests were considered significant at the *P* < 0.05 level.

## Results

### Patients

A total of 40 patients (28 males, 12 females, mean age: 65.2 ± 6.7 years) with carotid atherosclerotic plaques were included in the study. 20 patients (40 carotid arteries) in the asymptomatic group and 20 patients (40 carotid arteries) in the symptomatic group were involved in the carotid morphology and plaque component analysis. There were no significant differences in gender, age, body mass index (BMI), hypertension, coronary heart disease, diabetes mellitus, blood lipid and homocysteinemia between the two groups (all *P* > 0.05). The levels of fasting blood glucose, hs-CRP and glycosylated hemoglobin in the symptomatic group were significantly higher than the asymptomatic group (*P* < 0.05) (Table 2).

**TABLE 2:** Patients population.

	Asymptomatic	Symptomatic	*P-*value
Male (%)	12 (60.0)	16 (80.0)	0.243
Age	63.1 ± 4.4	66.1 ± 4.8	0.342
BMI(kg/m^2^)	24.5 ± 4.3	24.5 ± 3.8	0.573
Hypertension (%)	12 (60.0)	16 (80.0)	0.412
Coronary heart disease (%)	4 (20.0)	6 (30.0)	0.134
Diabetes (%)	8 (40.0)	13 (65.0)	0.168
Smoking (%)	15 (75.0)	18 (90.0)	0.172
Low density lipoprotein (mmol/L)	2.73 ± 0.47	2.84 ± 0.69	0.735
High density lipoprotein (mmol/L)	1.17 ± 0.31	1.32 ± 0.41	0.658
Total cholesterol (mmol/L)	3.82 ± 0.83	4.37 ± 0.85	0.547
Triglyceride (mmol/L)	1.12 ± 0.34	1.63 ± 0.42	0.672
Fasting blood glucose (mmol/L)	5.14 ± 1.02	5.92 ± 1.16	0.036*
Homocysteine (mmol/L)	12.73 ± 2.82	15.82 ± 3.01	0.123
High sensitivity C-reactive protein (mg/L)	1.32 ± 0.21	3.21 ± 0.42	0.015*
Glycosylated hemoglobin (%)	5.73 ± 0.48	6.46 ± 0.54	0.032*

*Compared with the asymptomatic and symptomatic group, P < 0.05.

### Morphology of carotid atherosclerotic plaques on HR-MRI

The MRI image quality of all the 40 patients met the research requirements. A total of 71 plaques were found in 40 patients with carotid atherosclerotic plaques [AHA type I-II (15.5%); type III (18.3%); type IV-V (23.9%); type VI (32.4%); type VII (9.9%)]. While AHA type VI was the most common type (23, 32.4%). A total of 864 axial MR image sections and classified according to the morphological characteristics of plaque, AHA type II-III accounted for 154 slices; type IV-V accounted for 278 slices; type VI accounted for 64 slices; type VII accounted for 109 slices. We found that carotid bifurcation was the most common lesion location (32, 45.1%) (Table 3).

**TABLE 3:** Distribution of MRI classification of carotid atherosclerotic plaques.

Location	I-II	III	IV-V	VI	VII	Total
Common carotid artery	2	0	4	3	2	11
carotid bifurcation	3	5	8	13	3	32
Internal carotid artery	6	8	5	7	2	28
Total	11	13	17	23	7	71

**FIGURE 2:**
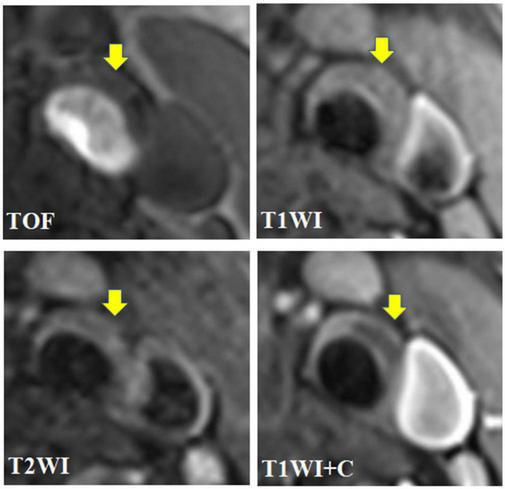
A 67 year-old male presented with intermittent dizziness for 2 weeks. The transverse axial TOF-MRA showed mild stenosis of left common carotid artery and bifurcation with local plaque formation, and the plaque was isointense; HR-MRI axial T1WI showed that the left carotid plaque was isointense; HR-MRI axial T2WI showed low signal in the left carotid plaque and isosignal on the plaque surface; HR-MRI axial T1WI enhancement showed that the surface of left carotid artery plaque showed obvious zonal enhancement, but no enhancement was found in the plaque; To sum up, the plaque components of the left common carotid artery and its bifurcation are the core of lipid necrosis, and the surface fiber cap is intact, which belongs to AHA type V.

**FIGURE 3:**
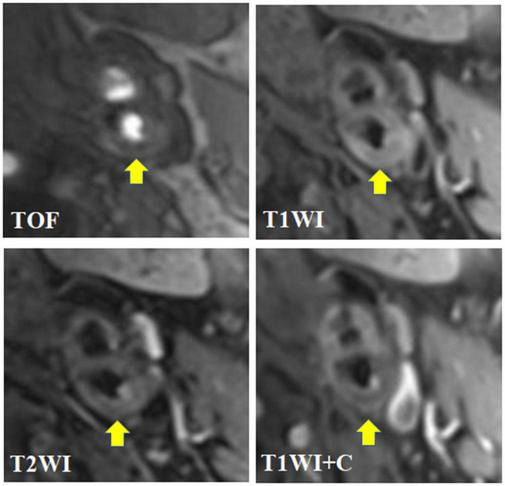
A 62-year-old male presented with dizziness and numbness of right limbs for more than 1 month. The transverse axial TOF-MRA showed moderate stenosis in the initial segment of the left internal carotid artery with the formation of centripetal plaques. The plaques were mainly slightly low signal with a small amount of high signal; HR-MRI transverse axial T1WI showed that the right internal carotid artery plaque was mainly slightly high signal, and slightly low signal shadow could be seen in it; HR-MRI transverse axis T2WI showed that the left internal carotid artery plaque was mainly slightly high signal, mixed with a small amount of low signal shadow, and the plaque surface showed irregular high signal; HR-MRI axial T1WI enhancement showed that the surface of left carotid artery plaque showed obvious interrupted linear enhancement, but no enhancement was found in the plaque; To sum up, the composition of plaques in the initial segment of the left internal carotid artery is complex, with visible lipid nuclei and calcification. The surface fiber cap rupture niche is formed with a small amount of bleeding (subacute), which belongs to AHA type VI, namely, vulnerable plaque.

In this study, carotid atherosclerotic plaques in MRI showed circular or eccentric wall thicken, with or without local crescent or semilunar signal protruding into the lumen. As the components of each type of plaque were different, and could contain multiple components at the same time, MRI manifestations were different (Figures 2, 3).

In the symptomatic group, 38 carotid plaques were found. Among the modified MRI classifications, AHA VI type was the most common (20, 52.6%), intraplaque hemorrhage (12, 31.6%), and fibrous cap rupture (6, 15.8%) were more common. AHA type VI was more common in carotid plaques and numbers of lipid core, hemorrhage, thrombosis and fiber cap rupture were more in symptomatic group. Compared with asymptomatic group, plaque burden including mean wall area, maximum wall area, maximum wall thickness, normalized wall index (NWI), lumen stenosis, maximum necrotic core area and the maximum intra-plaque hemorrhage area of carotid atherosclerotic plaques increased in symptomatic group (Table 4).

**TABLE 4:** Morphology of carotid atherosclerotic plaques on high resolution MRI.

	Symptomatic	Asymptomatic	*P*-value
**Plaque load**			
Mean wall area (mm^2^)	46.65 ± 21.31	42.85 ± 20.34	0.038*
Mean lumen area (mm^2^)	21.43 ± 15.42	28.76 ± 15.20	0.317
Maximum wall area (mm^2^)	75.35 ± 26.82	66.79 ± 25.43	0.023*
Maximum wall thickness (mm)	5.53 ± 2.15	3.32 ± 1.13	0.021*
Normalized wall index (%)	70.38 ± 11.32	61.52 ± 10.21	<0.001*
Lumen stenosis (%)	58.3 ± 18.36	42.93 ± 15.12	0.037*
**Plaque composition**			
Maximum necrotic core area (mm2)	10.82 ± 2.32	1.24 ± 0.21	0.000*
Maximum bleeding area (mm2)	1.51 ± 0.22	0.0	0.023*
Maximum calcification area (mm2)	0.43 ± 0.11	0.7 ± 0.12	0.374

*Compared with the asymptomatic and symptomatic group, P < 0.05.

### ^18^F-FDG uptake in carotid arteries measured with PET

Among the carotid atherosclerotic plaques classified by different modified MRI, only the TBR value of AHA VI was higher, that is, the uptake of ^18^F-FDG was significantly increased (AHA type VI: SUVmax = 3.31 ± 1.13, TBR = 3.21 ± 1.04, *P* < 0.001), while there were no significant difference in other modified MRI types.

**FIGURE 4:**
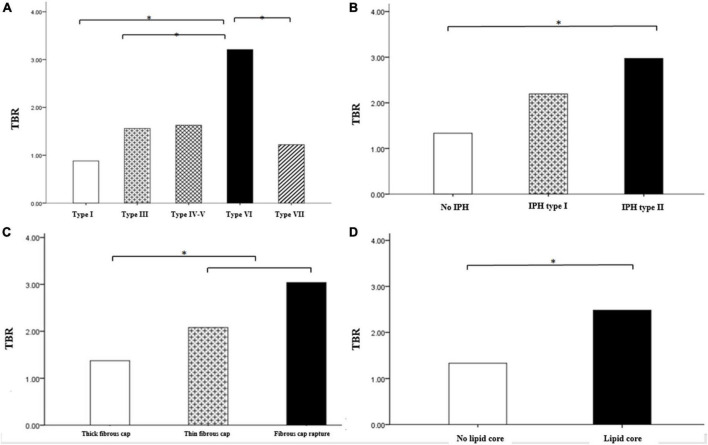
Comparison of TBR between carotid plaques of different MRI types. Comparison of TBR value of carotid plaques with different AHA classification **(A)** Comparison of TBR values of intra plaque hemorrhage **(B)** fiber cap state **(C)**, and lipid necrosis core **(D)**. Among the different types of carotid atherosclerotic plaques in the modified MRI classification, TBR values were higher in AHA type VI including intra-plaque hemorrhage, fibrous cap rupture and thin, also and lipid core. *P < 0.05.

**FIGURE 5:**
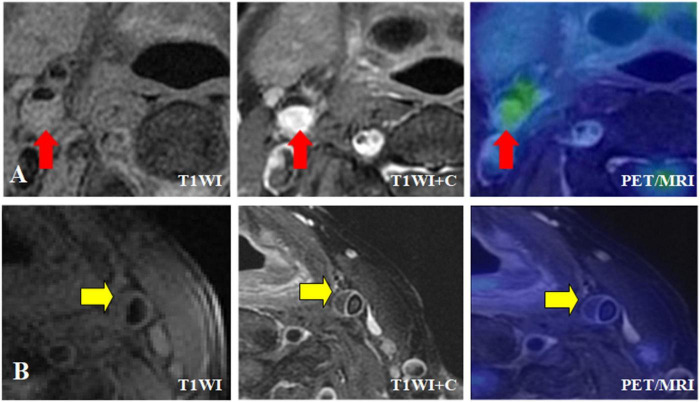
Different MRI types of carotid plaque integrated ^18^F-FDG PET/MR images. **(A)** Symptomatic patient with severe stenosis of right internal carotid artery lumen with plaque formation on T1WI, and the plaque was isointense. T1WI enhancement showed significant enhancement of right internal carotid artery plaque, and transverse fusion PET/MR showed high uptake of ^18^F-FDG, SUVmax = 3.5, TBR = 2.5. **(B)** Asymptomatic patient with moderate stenosis of left internal carotid artery with plaque formation, plaque showed equal signal, HR-MRI transverse axial T1WI showed obvious continuous band enhancement of left internal carotid artery plaque surface, no enhancement in plaque, transverse fusion PET/MR showed no uptake of plaque ^18^F-FDG, SUVmax = 2.05, TBR = 1.25.

In different MRI types of carotid atherosclerotic plaques, the TBR value with intra-plaque hemorrhage was higher, that is, the uptake of ^18^F-FDG was significantly increased (SUVmax = 2.17 ± 0.37, TBR = 2.11 ± 0.36, *P* < 0.05; SUVmax = 2.93 ± 0.54, TBR = 2.84 ± 0.53, *P* < 0.05). The TBR values of the patients with fiber cap rupture and thin fiber cap were higher, that is, the uptake of ^18^F-FDG was significantly increased (SUVmax = 2.07 ± 0.32, TBR = 2.01 ± 0.31, *P* < 0.05; SUVmax = 2.89 ± 0.54, TBR = 2.81 ± 45, *P* < 0.05) (Figures 4, 5). The TBR value of patients with lipid necrosis core was higher, that is, the uptake of ^18^F-FDG was significantly increased. TBR values of carotid plaque in symptomatic group (TBR = 2.56 ± 0.34) was significantly higher than that in asymptomatic group (TBR = 1.57 ± 0.14).

### Logistic regression analysis of risk factors of carotid plaque stability

Hypersensitive C-reactive protein was an independent risk factor for the stability of carotid artery plaque (*P* < 0.05), However, fasting blood glucose and glycosylated hemoglobin were not independent risk factors for the stability of carotid atherosclerotic plaque (*P* > 0.05) (Table 5).

**TABLE 5:** Logistic regression analysis of fasting blood glucose, hs-CRP and glycosylated hemoglobin among multiple groups.

	OR	95% CI	*P*-value
Fasting blood glucose	1.54	0.81−3.12	0.234
hs-CRP	3.65	1.87−6.54	<0.001*
Glycosylated hemoglobin	1.42	0.44−4.59	0.562

hs-CRP, hypersensitive C-reactive protein; OR, ratio; CI, confidence interval. *Logistic regression analysis among multiple groups, *P* < 0.05.

### Correlation analysis of multiple variables between symptom group and asymptomatic group

**FIGURE 6:**
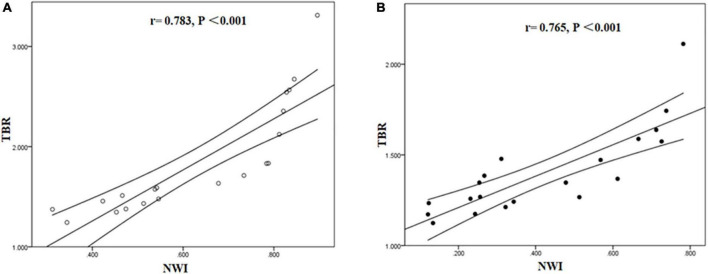
Correlation between NWI and TBR in symptomatic and asymptomatic patients. The correlation between NWI and TBR values was significantly positive in symptomatic **(A)** and asymptomatic group **(B)**.

The correlation between NWI and TBR values was significantly positive in symptomatic (*r* = 0.783, *P* < 0.001) and asymptomatic group (*r* = 0.765, *P* < 0.001) (Figure 6).

**FIGURE 7:**
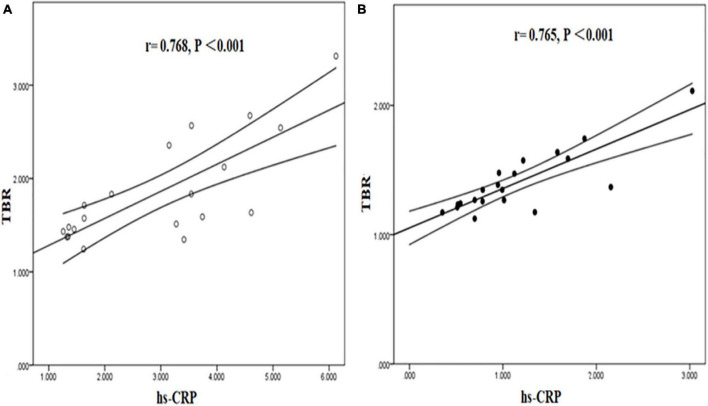
Correlation between hs-CRP and TBR in symptomatic and asymptomatic patients. The correlation between hs-CRP and TBR values was significantly positive in symptomatic **(A)** and asymptomatic group **(B)**.

**FIGURE 8:**
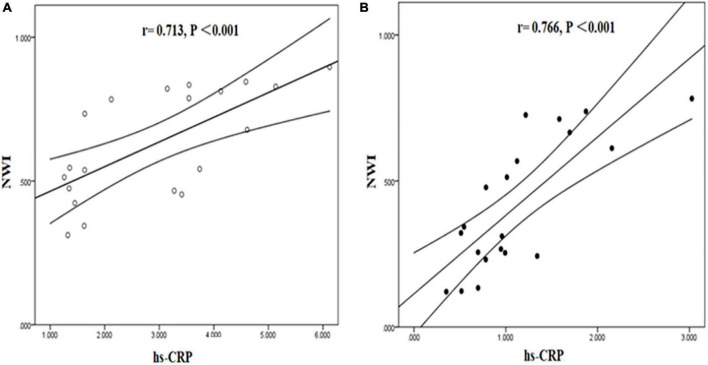
Correlation between hs-CRP and NWI in symptomatic and asymptomatic patients. The correlation between hs-CRP and NWI values was significantly positive in symptomatic **(A)** and asymptomatic group **(B)**.

Tissue to background ratio and NWI were significantly positively correlated with hs-CRP in symptomatic and asymptomatic group. [TBR (symptomatic group: *r* = 0.768, *P* < 0.001; asymptomatic group: *r* = 0.765, *P* < 0.001), NWI (symptomatic group: *r* = 0.713, *P* < 0.001; asymptomatic group: *r* = 0.766, *P* < 0.001)] (Figures 7, 8).

## Discussion

Plaque rupture in the carotid artery is the main cause of ischemic stroke and seriously threatens human health. Therefore, early identification of vulnerable plaques and evaluation of plaque stability is of great value for clinical early warning and improvement of quality of life. HR-MRI can accurately provide morphological information of plaque components, but cannot accurately assess plaque inflammatory response. PET, as a molecular imaging technique, can quantify macrophage inflammation in plaques, but it is limited by its poor spatial resolution and tissue resolution. Combined HR-MRI with PET can accurately assess carotid plaque vulnerability by evaluating both plaque morphology and Inflammatory cell metabolism (Saito et al., 2013). This study highlighted the potential of PET/MR as a non-invasive imaging tool for investigating morphological and inflamed-metabolism features of carotid atherosclerotic plaques between symptomatic and asymptomatic patients, such that vulnerable plaques may be distinguished from lower risk plaques for patient stratification and management in the clinic.

Firstly, PET/MR can classify carotid atherosclerotic plaque based on HR-MRI. In the study of the carotid plaque occurrence site, there were statistical differences in the distribution of different types of carotid artery plaque, and the plaque was more likely to occur in the bifurcation of the common carotid artery. This may be related to the hemodynamics of the carotid bifurcation (Figueroa et al., 2012). Due to the anatomical structure of the carotid bifurcation, a certain angle is formed here. The intima is more fragile than other places, and a series of complex compensatory changes such as endothelial cell damage and increased permeability can easily lead to atherosclerotic lesions in arteries. According to the classification of atherosclerotic plaques based on intraplaque fibrous caps and different vascular components, Cai et al. (2002, 2005) revised the plaque classification of AHA using HR MRI for the diagnosis of atherosclerotic plaque. In this study, the modified version of the AHA classification standard based on MRI was used for the classification of carotid artery plaques. In the MRI classification of carotid artery plaques, the components of type IV-V plaques and type VI plaques are vulnerable and related to the occurrence of ischemic stroke, especially type VI plaques, which frequently form emboli and cause distal vascular occlusion, thereby promoting the occurrence of cerebral ischemic events.

In the comparison of the morphological quantitative indicators of carotid atherosclerotic plaque between the symptomatic group and the asymptomatic group, our study found that the mean wall area, maximum wall area, maximum wall thickness, NWI, and lumen stenosis of the carotid artery in the symptomatic group were statistically greater than those in the asymptomatic group in terms of carotid plaque burden. It suggested that these indicators may have value in predicting the occurrence of stroke events. NWI was greater in the symptomatic group than in the asymptomatic group, suggesting that the standardized wall index is a valuable indicator for assessing plaque burden. NWI may also be used as an indicator to measure the dynamic changes of the vessel wall. This study found that the maximum lipid necrosis core area and the maximum hemorrhage area of carotid plaques in the symptomatic group were larger than those in the asymptomatic group, indicating that there were more vulnerable plaques in the symptomatic group.

Secondly, ^18^F-FDG PET/MR revealed ^18^F-FDG uptake and inflammatory response in different types of carotid atherosclerotic plaques. Studies found that the surgically resected plaque contained a large number of macrophages in the area around the carotid artery of a TIA patient with a stenosis of 70%, and the uptake of components by ^18^F-FDG was related to the density of macrophages (Rudd et al., 2002; Davies et al., 2010; Ng et al., 2023). There is a close relationship between macrophages and ^18^F-FDG uptake (Ripa et al., 2013; Mootaz et al., 2015). Therefore, PET/MR can provide morphological structure and molecular metabolism information of plaques, clarify the inflammatory response mechanism in different types of plaques, and accurately evaluate the stability of plaques.

In this study, according to the plaque components of HR-MRI images and the TBR value, there are significant differences in the inflammatory response among different types of carotid atherosclerotic plaque components. The TBR value of the plaques with intraplaque hemorrhage and the plaques with lipid necrosis core was higher than that of other plaques, and the TBR value of the plaques with calcification was the lowest. The reason may be that intraplaque hemorrhage is mainly the rupture of new capillaries, which leads to the leakage of blood into the plaque to form a hematoma (Rudd et al., 2002; Ng et al., 2023). The increase of lipid necrotic core in plaque will accelerate plaque formation. Our study found that the proportion of lipid core in carotid artery plaques in the symptomatic group was significantly higher than that in the asymptomatic group, suggesting that the lipid necrotic core was related to the stability of the plaque. The study also found that the TBR value of carotid plaque with a rich lipid core was higher, which suggested that the lipid necrosis core could be used as the characteristic of vulnerable carotid plaque, which was consistent with the previous study (Cai et al., 2002, 2005).

Thirdly, this study showed the clinical value of high-sensitivity C-reactive protein in carotid artery vulnerability. As an acute response protein and an inflammatory marker, C-reactive protein (CRP) can respond to infection, inflammation, and tissue damage, and its plasma concentration rises rapidly, even up to 10,000 times (Black et al., 2004; Newby, 2005). The new CRP test method further improved the sensitivity of detection. Previous studies have shown that CRP accelerates the formation and progression of atherosclerosis (Black et al., 2004; Newby, 2005; Clarke et al., 2006). Elevated plasma CRP concentrations are associated with carotid intima-media thickening, the occurrence, development, and rupture of sclerotic plaques, and subsequent cerebrovascular events in patients with or without symptoms of carotid stenosis (Sproston and Ashworth, 2018; Yao et al., 2019). This study also found that the hs-CRP of carotid plaques in the symptomatic group was significantly higher than that of the asymptomatic plaques, and was significantly positively correlated with the TBR value and NWI of carotid plaques, suggesting that hs-CRP is also involved in plaque inflammation reaction.

CRP is an acute reactive protein and inflammatory marker, which can accelerate the formation and progression of atherosclerosis, thus aggravating the plaque load (Black et al., 2004; Newby, 2005). Meanwhile, this pathological process also increases NWI and TBR, while the former is an important indicator to evaluate the plaque load and the latter is a quantitative indicator to evaluate the plaque inflammatory response (Ripa et al., 2013). In the whole process of atherosclerosis, the interaction between these multi-factors will further accelerate its progress. Therefore, serum hs-CRP can be used as an independent risk factor reflecting the instability of atherosclerotic plaque, which has a certain predictive value and is beneficial to early clinical prevention.

In this study, the hybrid ^18^F-FDG PET/MR revealed the difference of ^18^F-FDG uptake of different carotid atherosclerotic plaque components, combined with clinical serum hs-CRP indicator, so as to quantitatively evaluate the characteristics and stability of carotid atherosclerotic plaque and provide objective imaging basis for clinical early warning of ischemic stroke. Implication of vulnerable plaque in asymptomatic patients *via* ^18^F-FDG PET/MR can guide early diagnosis and medical intervention in the clinic to effectively prevent stroke.

On the limitations, our results were limited by the small sample size of patients with different clinical symptoms. In addition, the pathophysiological mechanism of the inflammatory response process, which could be closely related to the occurrence, development, and rupture of plaques, should be further explored using ^18^F-FDG PET/MR.

## Conclusion

This study had demonstrated that morphological and inflamed-metabolism features of carotid atherosclerotic plaques can be evaluated with the integrated ^18^F-FDG PET/MR system. hs-CRP could be regarded as an independent risk factor for the stability of carotid atherosclerotic plaque. Hybrid ^18^F-FDG PET/MR system combined with clinical serum hs-CRP indicator might help distinguish vulnerable carotid plaque from lower risk plaques and that provides early warnings for clinical management.

## Data availability statement

The raw data supporting the conclusions of this article will be made available by the authors, without undue reservation.

## Ethics statement

The studies involving human participants were reviewed and approved by the Ethics Committee of Xuanwu Hospital. The patients/participants provided their written informed consent to participate in this study.

## Author contributions

JL and YZ: conceptualization. YZ: writing—original draft. YZ, JL, and XL: review and revision. BC, HY, JM, BY, YM, and LJ: experiment and data collection. All authors contributed to the article and approved the submitted version.
