# Corrigendum: Morphological feature and mapping inflammation in classified carotid plaques in symptomatic and asymptomatic patients: a hybrid ^18^F-FDG PET/MR study

**DOI:** 10.3389/fnins.2023.1209830

**Published:** 2023-05-10

**Authors:** Yue Zhang, Bixiao Cui, Hongwei Yang, Jie Ma, Yu Yang, Bin Yang, Yan Ma, Liqun Jiao, Xiang Li, Jie Lu

**Affiliations:** ^1^Department of Radiology and Nuclear Medicine, Xuanwu Hospital, Capital Medical University, Beijing, China; ^2^Beijing Key Laboratory of Magnetic Resonance Imaging and Brain Informatics, Beijing, China; ^3^Department of Neurosurgery, Xuanwu Hospital, Capital Medical University, Beijing, China; ^4^Division of Nuclear Medicine, Department of Biomedical Imaging and Image-Guided Therapy, Medical University of Vienna, Vienna, Austria

**Keywords:** hybrid ^18^F-FDG PET/MR, atherosclerosis, vulnerable plaque, carotid arteries, symptomatic, asymptomatic

In the published article, there was an error in Patients population of Table 2 as published. There was some missing information in Table 2 during the process of uploading the Table 2. The corrected Patients population of Table 2 and its caption ^*^ [Note: Compared with the asymptomatic and symptomatic group, *P* < 0.05.] appear below.

**Table 2:** Patients population.

	**Asymptomatic**	**Symptomatic**	***P*** **value**
Male (%)	12 (60.0)	16 (80.0)	0.243
Age	63.1 ± 4.4	66.1 ± 4.8	0.342
BMI(kg/m^2^)	24.5 ± 4.3	24.5 ± 3.8	0.573
Hypertension (%)	12 (60.0)	16 (80.0)	0.412
Coronary heart disease (%)	4 (20.0)	6 (30.0)	0.134
Diabetes (%)	8 (40.0)	13 (65.0)	0.168
Smoking (%)	15 (75.0)	18 (90.0)	0.172
Low density lipoprotein (mmol/L)	2.73 ± 0.47	2.84 ± 0.69	0.735
High density lipoprotein (mmol/L)	1.17 ± 0.31	1.32 ± 0.41	0.658
Total cholesterol (mmol/L)	3.82 ± 0.83	4.37 ± 0.85	0.547
Triglyceride (mmol/L)	1.12 ± 0.34	1.63 ± 0.42	0.672
Fasting blood glucose (mmol/L)	5.14 ± 1.02	5.92 ± 1.16	0.036^*^
Homocysteine (mmol/L)	12.73 ± 2.82	15.82 ± 3.01	0.123
High sensitivity C-reactive protein (mg/L)	1.32 ± 0.21	3.21 ± 0.42	0.015^*^
Glycosylated hemoglobin (%)	5.73 ± 0.48	6.46 ± 0.54	0.032^*^

* Compared with the asymptomatic and symptomatic group, P < 0.05.

In the published article, there was an error in Morphology of carotid atherosclerotic plaques on high resolution MRI of Table 4 as published. The positions of the symptomatic and asymptomatic groups in Table 4 were reversed. The corrected Morphology of carotid atherosclerotic plaques on high resolution MRI of Table 4 and its caption ^*^ [Note: Compared with the asymptomatic and symptomatic group, *P* < 0.05.] appear below.

**Table 4:** Morphology of carotid atherosclerotic plaques on high resolution MRI.

	**Symptomatic**	**Asymptomatic**	***P*** **-value**
**Plaque load**			
Mean wall area (mm^2^)	46.65 ± 21.31	42.85 ± 20.34	0.038^*^
Mean lumen area (mm^2^)	21.43 ± 15.42	28.76 ± 15.20	0.317
Maximum wall area (mm^2^)	75.35 ± 26.82	66.79 ± 25.43	0.023^*^
Maximum wall thickness (mm)	5.53 ± 2.15	3.32 ± 1.13	0.021^*^
Normalized wall index (%)	70.38 ± 11.32	61.52 ± 10.21	< 0.001^*^
Lumen stenosis (%)	58.3 ± 18.36	42.93 ± 15.12	0.037^*^
**Plaque composition**			
Maximum necrotic core area (mm^2^)	10.82 ± 2.32	1.24 ± 0.21	0.000^*^
Maximum bleeding area (mm^2^)	1.51 ± 0.22	0.0	0.023^*^
Maximum calcification area (mm^2^)	0.43 ± 0.11	0.7 ± 0.12	0.374

* Compared with the asymptomatic and symptomatic group, P < 0.05.

The authors apologize for this error and state that this does not change the scientific conclusions of the article in any way. The original article has been updated.

